# Hsp70 Architecture: The Formation of Novel Polymeric Structures of Hsp70.1 and Hsc70 after Proteotoxic Stress

**DOI:** 10.1371/journal.pone.0052351

**Published:** 2012-12-19

**Authors:** Rohan Steel, Ryan S. Cross, Sarah L. Ellis, Robin L. Anderson

**Affiliations:** 1 Peter MacCallum Cancer Centre, St Andrew’s Place, East Melbourne, Victoria, Australia; 2 The Sir Peter MacCallum Department of Oncology, The University of Melbourne, Parkville, Victoria, Australia; Boston University Medical School, United States of America

## Abstract

Heat induces Hsp70.1 (HSPA1) and Hsc70 (HSPA8) to form complex detergent insoluble cytoplasmic and nuclear structures that are distinct from the cytoskeleton and internal cell membranes. These novel structures have not been observed by earlier immunofluorescence studies as they are obscured by the abundance of soluble Hsp70.1/Hsc70 present in cells. While resistant to detergents, these Hsp70 structures display complex intracellular dynamics and are efficiently disaggregated by ATP, indicating that this pool of Hsp70.1/Hsc70 retains native function and regulation. Hsp70.1 promotes the repair of proteotoxic damage and cell survival after stress. In heated fibroblasts expressing Hsp70.1, Hsp70.1 and Hsc70 complexes are efficiently disaggregated before the cells undergo-heat induced apoptosis. In the absence of Hsp70.1, fibroblasts have increased rates of heat-induced apoptosis and maintain stable insoluble Hsc70 structures. The differences in the intracellular distribution of Hsp70.1 and Hsc70, combined with the ability of Hsp70.1, but not Hsc70, to promote the disaggregation of insoluble Hsp70.1/Hsc70 complexes, indicate that these two closely related proteins perform distinctly different cellular functions in heated cells.

## Introduction

Hsp70.1 (HSPA1) and Hsc70 (HSPA8) are members of the Hsp70 family of proteins that are present in the cytoplasm and nucleus of all mammalian cells [1]. They have been shown to participate in diverse processes including the folding of newly translated proteins [2], protein trafficking across intracellular membranes [3] and the assembly/disassembly of protein complexes [4], [5]. Hsp70.1 and Hsc70 share 84% amino acid identity [4], [5] and are often assumed to be functionally analogous, with only a few reports suggesting different functions [6], [7], [8].

Hsp70.1 has a critical role in protecting cells from stress and protein damage, a process known as thermotolerance [6]. The increased resistance to cell death in thermotolerant cells may be related to the ability of Hsp70.1 to repair protein damage [7], [8]. While protein repair is clearly important for cell viability, Li et al found that the ability of Hsp70.1 to inhibit cell death is distinct from its ability to repair transcription and translation in heated cells [9]. It has been proposed that Hsp70.1 inhibits apoptosis by interacting directly with the apoptotic signaling machinery as it can also inhibit apoptosis in response to stresses that do not cause protein damage [10], [11], [12], [13]. Proposed targets for regulation of apoptosis by Hsp70.1 include JNK/SAPK, Bax and the apoptosome [14], [15], [16], [17]. However subsequent studies have failed to confirm these proteins as the sites of Hsp70.1 regulation [18], [19], [20]. Hsp70.1 has also been reported to inhibit apoptosis by inhibiting the release of cathepsins from lysosomes and by the degradation of Mcl-1, both by unknown mechanisms [18], [21]. Clearly, the identification of anti-apoptotic Hsp70.1 binding substrates is critical to understanding how Hsp70.1 protects cells from stress.

In this study we examined the interactions of Hsp70.1 and Hsc70 with insoluble intracellular structures that are normally discarded during the preparation of cytosolic extracts. Using MEF-H2 cells, a model cell line used to study stress induced apoptosis [10], we show that proteotoxic stress induces the formation of novel insoluble structures of Hsp70.1 and Hsc70 in the cytoplasm and nuclei of cells. Furthermore, the rapid disaggregation of these complexes correlates with cell survival after exposure to heat.

## Results

### Proteotoxic Stress Induces Hsp70.1 and Hsc70 Binding to Digitonin Resistant Cell Components

**Figure 1:**
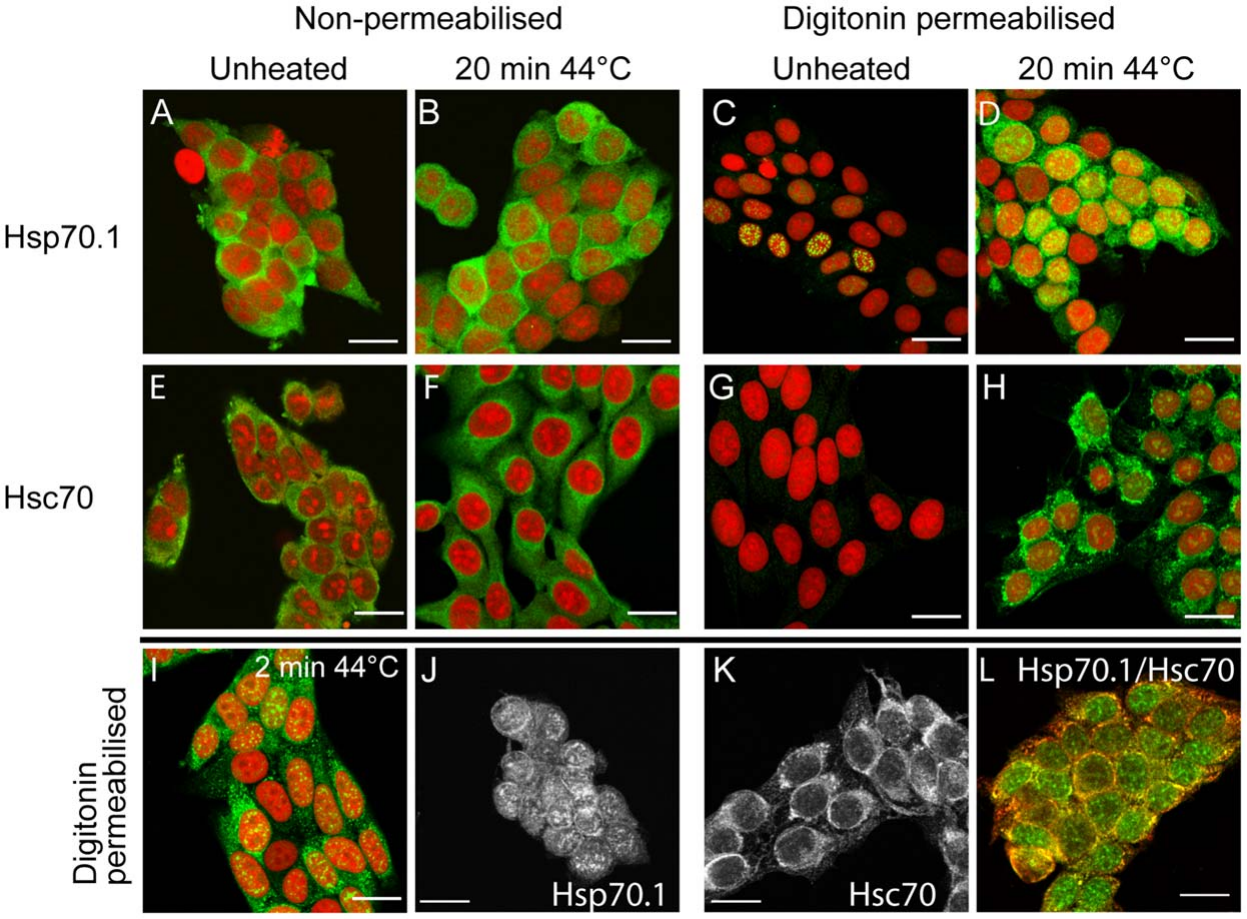
Proteotoxic stress induces Hsp70.1/Hsc70 binding to insoluble cell structures. MEF-H2 were maintained at 37°C (unheated) or heated at 44°C for 20 min and fixed immediately with 4% paraformaldehyde. Hsp70.1 and Hsc70 were detected by immunofluorescence (green) and the nuclei stained with PI (red): Hsp70.1, unheated (A); Hsp70.1, heated (B); Hsc70, unheated (E); Hsc70, heated (F). MEF-H2 were treated as described above but permeabilized with 100 µg/ml digitonin prior to fixation: Hsp70.1, unheated (C); Hsp70.1, heated (D); Hsc70, unheated (G); Hsc70, heated (H). MEF-H2 heated at 44°C for 2 min, permeabilized, Hsp70.1 staining (I); MEF-H2 cells heated at 44°C for 20 min, digitonin permeabilized and stained for Hsp70.1 (J) and Hsc70 (K); MEF-H2 heated at 44°C for 20 min, double stained for Hps70.1 (green) and Hsc70 (red) (L). Scale bars represent 20 µm.

Immunofluorescence has been used extensively to observe Hsp70.1 and Hsc70 in mammalian cells, but aside from the heat induced localization of these two proteins to the nucleus and nucleolus, immunofluorescence has revealed little detail of Hsp70 function [22], [23], [24]. In formaldehyde fixed MEF-H2 that constitutively express exogenous human Hsp70.1 [10], Hsp70.1 displayed a diffuse nuclear and cytoplasmic distribution, indicative of a soluble protein (Fig. 1A). Little change in Hsp70.1 distribution was observed immediately after heating, although there was a small increase in protein present in the nucleus (Fig. 1B).

To study Hsp70.1 interactions with insoluble cell components, MEF-H2 were permeabilized with digitonin prior to fixation, allowing soluble cytoplasmic proteins to diffuse out of the cell while leaving the internal cell membranes and the cytoskeleton intact [25]. In unheated MEF-H2, very little Hsp70.1 was detected after digitonin extraction (Fig. 1C), illustrating the efficiency of this procedure for removing soluble cytoplasmic proteins from the cells. Heating MEF-H2 at 44°C for 20 minutes caused a dramatic increase in Hsp70.1 binding to insoluble cell structures in the cytoplasm and the nucleus (Fig. 1D). Heating MEF-H2 for only 2 minutes at 44°C, a non-lethal heat dose (data not shown) produced similar levels of insoluble Hsp70.1 fluorescence, indicating the sensitivity of Hsp70.1 to heat induced damage (Fig. 1I).

A similar result was obtained for the cognate Hsc70 protein. Immunofluorescence of non-permeabilized cells revealed a diffuse staining pattern restricted to the cytoplasm (Fig. 1E) that contracted slightly to the perinuclear region after heating (Fig. 1F). Digitonin extraction removed almost all detectable Hsc70 from the cytoplasm in unheated cells (Fig. 1G) while heat induced the accumulation of insoluble cytoplasmic Hsc70 (Fig. 1H).

Unlike the diffuse pattern observed in non-permeabilized cells, the Hsp70.1 and, in particular, Hsc70 detected in permeabilized cells after heat was present in a complex pattern, with the appearance of a fine polymeric network (Fig. 1J & K). The differential distribution of Hsp70.1 and Hsc70 was confirmed in permeabilized MEF-H2, heated at 44°C for 20 min and then immunostained for both Hsp70.1 (green) and Hsc70 (red). Hsp70.1 and Hsc70 co-localize to the cytoplasm while only Hsp70.1 is located in the nucleus (Fig. 1L).

The heat induced binding of Hsp70.1 and Hsc70 to insoluble intracellular structures is not unique to MEF. Heat induced a similar response in HEK293 cells that express naturally high levels of Hsp70.1 (Fig. S1 A & B) and in L929 cells (data not shown). Nor is it restricted to heat. MEF-H2 treated with sodium arsenite also displayed Hsp70.1 binding to insoluble cell structures (Fig. S1C & D). In comparison, non-proteotoxic forms of stress such as TNFα, UV radiation and etoposide did not induce the aggregation of Hsp70.1/Hsc70 (data not shown).

### The Binding of Hsp70.1 and Hsc70 to Insoluble Cell Structures is Reversed by ATP

**Figure 2:**
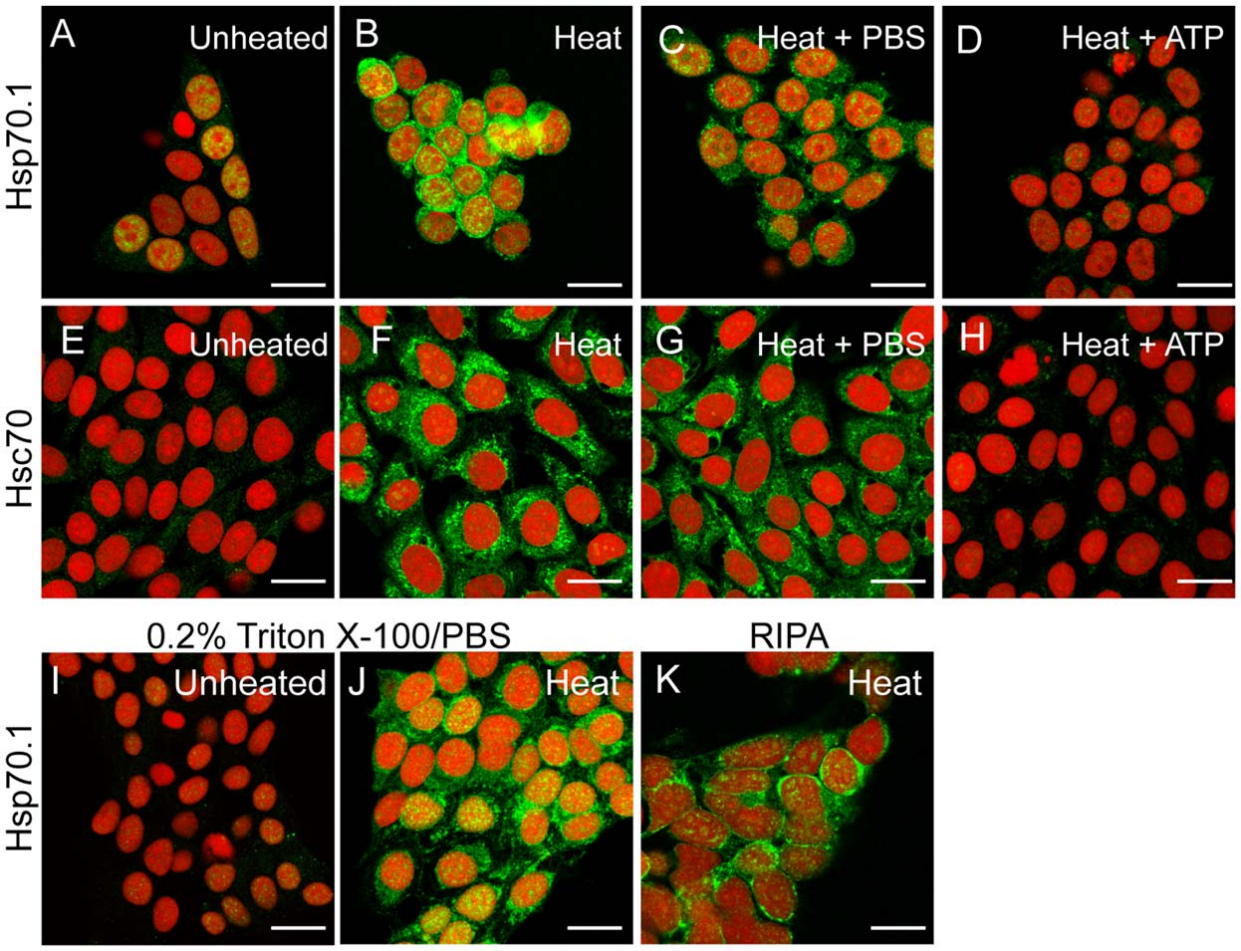
Hsp70.1/Hsc70 complexes are disassociated by ATP but not by detergent. MEF-H2 stained for Hsp70.1 (A, B, C, D, I, J, K) or Hsc70 (E, F, G, H) (green). Nuclei stained with PI (red). All heated cells were incubated at 44°C for 20 min: unheated/digitonin permeabilized (A, E); heated/digitonin permeabilized (B, F); heated/digitonin permeabilized/incubated with PBS at room temp for 10 min (C, G); heated/digitonin permeabilized/incubated with 2 mM ATP at RT for 10 min (D, H). Unheated MEF-H2 permeabilized with 0.2% TX100 (I); heated and permeabilized with 0.2% TX100 (J); heated and permeabilized with RIPA (K). Scale bars represent 20 µm.

The binding of Hsp70.1 and Hsc70 to protein substrates via the peptide-binding domain is inhibited by ATP [26]. As shown in Fig. 2A–H, the incubation of digitonin permeabilized MEF-H2 with 2 mM ATP efficiently extracted the immobilized Hsp70.1 and Hsc70 (Fig. 2 D & H). Incubating cells in PBS under identical conditions had little impact on the binding of both proteins although some degradation of the internal cell structures was observed (Fig. 2 C & G). The efficient extraction of Hsp70.1 and Hsc70 by ATP confirms that the aggregation of Hsp70.1/Hsc70 is dependent on interactions involving the substrate binding domain, regulated by the ATP binding domain, and is not due merely to Hsp70.1/Hsc70 denaturation.

### Heat Induced Hsp70.1/protein Complexes are Resistant to Detergent Extraction

Identifying the heat induced binding substrates of Hsp70.1 and Hsc70 raises technical challenges when the binding targets are insoluble intracellular structures. To determine if the binding substrates were membrane or organelle associated, MEF-H2 were treated with detergents to remove all membranes and membrane associated proteins, in addition to the soluble cytosolic proteins extracted by digitonin. Unheated MEF-H2 treated with 0.2% Triton X-100 (TX100) contained no detectable Hsp70.1 (Fig. 2I), removing the traces of Hsp70.1 still detected in digitonin treated cells (Fig. 2A). Heated MEF-H2 cells extracted with TX100 (Fig. 2J) displayed an Hsp70.1 binding pattern identical to cells permeabilized with digitonin (Fig. 2B). Even cells extracted with a more stringent RIPA buffer showed significant retention of Hsp70.1 in the cytoplasm (Fig. 2K), indicating that Hsp70.1 was associated with a proteinaceous structure and not associated with intracellular membranes.

### Hsp70.1 and Hsc70 do not Associate with Cytoskeletal Structures

Since Hsp70.1 and Hsc70 were not associated with membrane structures, we concluded that the two proteins were bound to a structural protein network, possibly associated with the cytoskeleton. This conclusion was supported by the fibrillar appearance of the insoluble proteins in digitonin permeabilized cells (Fig. 1 J&K), particularly by the distribution of Hsc70 (Fig. 1K). There have been previous reports of Hsp70.1 and Hsc70 associating with cytoskeletal proteins and thermotolerant cells have been shown to be resistant to heat induced cytoskeletal changes [27]. However, the Hsp70.1 and Hsc70 structures observed in heated and digitonin extracted cells were distinctly different from actin and tubulin networks present in the same cells (Fig. S2 A & B). Nor did Hsp70.1 or Hsc70 co-localize with vimentin in either heated or non-heated cells (Fig. S2 C–F) or with the proteasome (data not shown). As vimentin and the proteasome are common components of aggresomes, it does not appear that the novel Hsp70.1/Hsc70 structures are a part of the cellular response to aggregated protein [28]. Electron microscopy of formaldehyde fixed cells also failed to identify the structures formed by Hsp70.1/Hsc70 in heated cells (data not shown).

### Hsp70.1 and Hsc70 do not Associate with Stress Granules

Stress granules appear in the cytoplasm of cells after a variety of stresses, including heat. They contain mRNAs for many proteins, but exclude mRNA of heat shock proteins and act to arrest protein synthesis in stressed cells, thereby preventing protein misfolding [29]. Given that the Hsp70.1/Hsc70 structures we have observed are also present in the cytoplasm after stress, we asked if the digitonin insoluble Hsp70.1 complexes were associated with stress granules. Using eIF4E as a marker of stress granules [30], co-localization of HSP70.1 with eIF4E in MEF-H2 cells was assessed before and 2 h after exposure to 44°C for 20 min. As shown in Fig. S3, eIF4E has a diffuse cytoplasmic distribution in non-heated cells and is released from digitonin permeabilized cells, as is HSP70.1. After heat and digitonin permeabilization, eIF4E positive stress granules are evident, but display a very different distribution to the insoluble HSP70.1 structures (Fig. S3). We therefore conclude that the polymeric HSP70 structures are not stress granules.

### Heat Induces the Aggregation of Hsp40 and HspBP1 but not Hsp90, Hip or Bag-1

**Figure 3:**
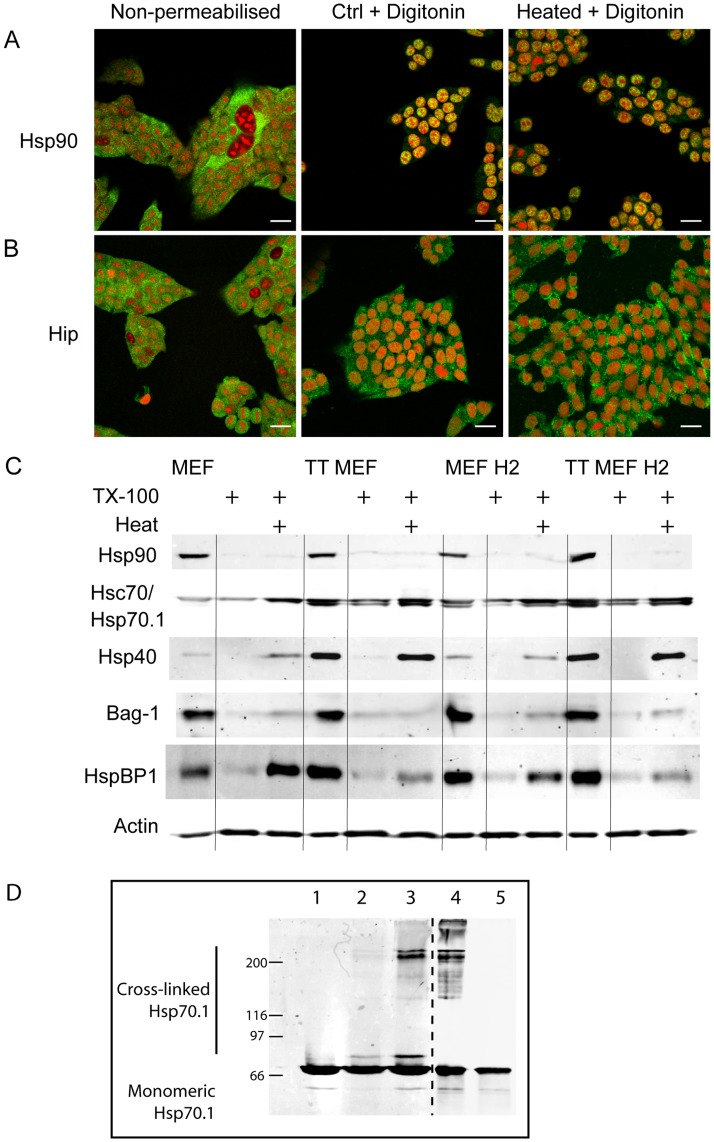
Heat induced association of Hsp70.1/Hsc70 with heat shock proteins and co-chaperones. A & B: heat induced binding of Hsp90 and Hip to insoluble cell structures in MEF-H2. Immunofluorescence of MEF-H2 cells stained for Hsp90 (A) and Hip (B). Immunostained proteins are green, nuclei stained with PI are red. Cells were heated at 44°C 20 min and permeabilized with 100 µg/ml digitonin. Scale bars represent 20 µm. C: Western blotting of TX100 insoluble proteins. Total cell proteins or TX100 insoluble proteins were analysed by SDS-PAGE and immunoblotted for Hsp90, Hsp70.1/Hsc70, Hsp40, Bag-1 and HspBP1. Cells were heated at 44°C for 20 min. D: Chemical crosslinking of heated & permeabilized cells. MEF-H2 were heated, extracted with 100 µg/ml digitonin and treated with DSS. Proteins were analysed by SDS-PAGE and immunoblotted for Hsp72 (lanes 1–5) Lane 1: heated cells no DSS, 2: unheated cells+DSS, 3: heated cells+DSS, 4: purified Hsp70.1+ DSS, 5: purified Hsp70.1 no DSS. This blot was scanned at two different settings to account for differences in signal intensity.

To determine if the heat induced insoluble Hsp70.1 and Hsc70 structures contained only these two proteins or included other heat shock proteins and co-chaperones, MEF-H2 were heated and permeabilized with digitonin before being probed with antibodies against Hsp90 and the co-chaperone Hip. It has been proposed that Hsp70.1 disaggregates denatured proteins and passes them to Hsp90 for further folding [31], while Hip is a known Hsp70.1 binding protein that also associates with Hsp90 [32]. Hsp90 was retained inside the nucleus of digitonin permeabilized cells (Fig. 3A) while some Hip was retained in the cytoplasm (Fig. 3B). In both cases, heat caused no visible changes in the distribution of Hsp90 or Hip in digitonin permeabilized cells, either immediately after heating or for up to 8 hours afterwards (data not shown).

The presence of other co-chaperones that act to regulate Hsp70.1/Hsc70 activity was examined by a combination of cell fractionation and western blotting, due to a lack of antibodies suitable for immunofluorescence. Soluble and membrane bound proteins were extracted from control and thermotolerant MEF and MEF-H2 with 0.2% TX100 and the remaining proteins were solubilized with Laemmli gel sample buffer and analyzed by western blotting.

Hsp90 was retained inside the nucleus in digitonin extracted cells (Fig. 3A) but in TX100 treated cells, where the nuclear membrane had been dissolved with detergent, Hsp90 was completely extracted in both heated and unheated cells (Fig. 3C). In contrast, there was a dramatic increase in the amount of TX100 insoluble Hsp70.1 and Hsc70 present in heated cells (Figure 3C), as observed previously by Kampinga et al. [33]. This analysis by cell fractionation confirms the results obtained previously by immunofluorescence.

Bag-1 is a co-chaperone known to bind to the N-terminal ATPase domain of Hsp70.1/Hsc70 [34]. Very little Bag-1 was found to associate with the TX100 insoluble cell fraction of heated cells (Fig. 3C). In contrast, two other co-chaperones, Hsp40 [35] and HspBP1 [36], were found to be present in the TX100 insoluble fraction after heat. Hsp40 is present in only trace quantities in MEF and MEF-H2 but is expressed abundantly in thermotolerant cells. Consequently, much higher levels of Hsp40 are retained in thermotolerant cells. HspBP1 is present in all cells but its heat induced binding to TX100 insoluble structures is inhibited in thermotolerant cells. The displacement of HspBP1 from Hsp70.1 by Hsp40 has been reported previously [37]. While it is possible that Hsp40 and HspBP1 may be associating with the insoluble components of heated cells independent of Hsp70.1/Hsc70, their presence is most likely linked to their function as Hsp70.1/Hsc70 co-chaperones. Hsp40 has already been shown to co-localize with Hsp70.1/Hsc70 in detergent permeabilized thermotolerant HeLa cells after heating [24].

### The Insoluble Hsp70.1 and Hsc70 form Polymeric Complexes

Current theories of Hsp70.1/Hsc70 function suggest that the two proteins should bind to heat denatured proteins. This may explain the insoluble Hsp70.1 and Hsc70 that we have observed in digitonin permeabilized cells. To identify the proteins bound by Hsp70.1, digitonin permeabilized cells were chemically cross-linked with disuccinimidyl suberate (DSS) before the proteins were solubilized in Laemmli gel sample buffer and examined by western blotting. When immunoblotted for Hsp70.1, any bands with molecular weights greater than 70 kDa will represent Hsp70.1 crosslinked to protein binding partners. As shown in Fig. 3D lane 3, three high molecular weight complexes containing Hsp70.1 were detected in heated cells after permeabilisation and cross-linking. Hsp70.1 crosslinking was much less in non-heated cells (lane 2) and no high molecular weight immunostained bands were detected in the absence of the cross-linking agent (lane 1). The limited number of Hsp70.1 complexes observed was surprising, as Hsp70.1 bound to denatured and aggregated proteins would be expected to produce many different complexes with varying electrophoretic mobility. A comparison with crosslinked purified Hsp70.1 (Fig. 3D lane 4), indicates that the two highest molecular weight bands represent Hsp70.1 homo-oligomeric complexes while the third unidentified band migrates just above the monomeric Hsp70.1. This lowest gel band could be due to an interaction with a small (∼5 kDa) protein. In the absence of crosslinking, purified Hsp70.1 runs at 70 kDa (lane 5). The homo-oligomerization of purified Hsp70.1 and Hsc70 has been demonstrated by previous studies [38]. Further immunoblotting for Hsp40 and HspBP1 did not reveal any high molecular weight bands co-migrating with Hsp70.1 or Hsc70 (data not shown).

### The Kinetics of Hsp70.1/Hsc70 Aggregation and Apoptosis in MEF and MEF-H2 Cells

**Figure 4:**
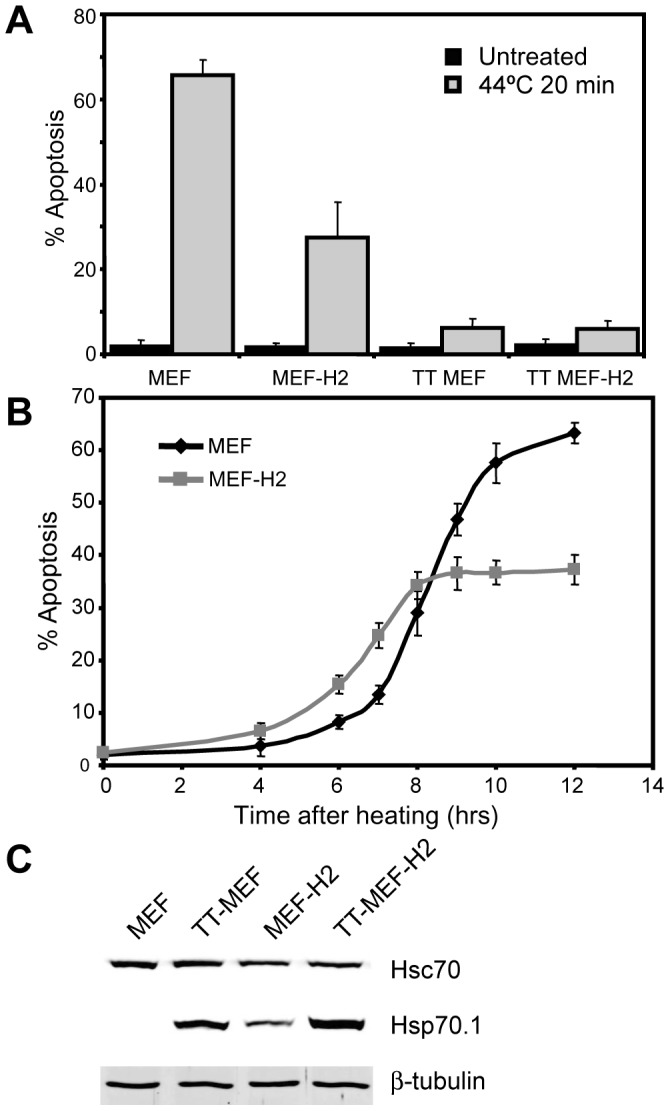
Kinetics of apoptosis in MEF and MEF-H2 after heat. Kinetics of apoptosis in MEF and MEF-H2. A: Heat induced apoptosis in MEF, MEF-H2, thermotolerant (TT) MEF and TT MEF-H2. Cells were heated at 44°C for 20 min and apoptosis scored by nuclear condensation after 16 hrs. Error bars represent SEM (N = 3). B: Kinetics of apoptosis. MEF and MEF-H2 were heated as above and apoptosis scored at specified times. Error bars represent SEM (N = 2, data from two experiments with triplicate samples). C: Total cell extracts from MEF, MEF-H2, TT MEF and TT MEF-H2 were immunoblotted for Hsc70 and Hsp70.1.

While Hsp70.1 and Hsc70 were found to form insoluble structures, it remained unclear how these structures might contribute to cell survival after heat shock. As reported previously, the expression of Hsp70.1 in MEF-H2 confers an increased resistance to heat induced apoptosis compared to parental MEF, while cells rendered thermotolerant by exposure to a non-lethal heat dose were almost completely resistant to heating at 44°C for 20 minutes (Fig. 4A) [10]. The kinetics of heat induced apoptosis revealed a highly regulated process, with apoptosis in MEF first detected at 6–8 hours after heating and almost all cells fated to undergo apoptosis having completed the process by 12 hours (Fig. 4B). MEF-H2 responded more rapidly with all heat-induced apoptosis completed by 8 hours after heat treatment. The basal level of Hsp70.1 and Hsc70 expression in the cells is shown in Fig. 4C.

To examine the kinetics of Hsp70.1/Hsc70 binding to insoluble cell structures after heat, normal and thermotolerant MEF and MEF-H2 were heated at 44°C for 20 minutes and harvested at 2 hourly intervals before being analyzed by immunofluorescence. To determine the total amount of Hsp70.1 present in cells, non-permeabilized cells were stained with anti-Hsp70.1 antibodies (Fig. S4). Thermotolerant (TT) MEF, MEF-H2 and TT MEF-H2 all expressed Hsp70.1 prior to heating while normal MEF did not express significant quantities of Hsp70.1 until 6 hours after heating. Despite the high levels of apoptosis induced in MEF by heating, surviving cells expressed levels of Hsp70.1 equivalent to the other cell lines 8 hours after heat treatment.

**Figure 5:**
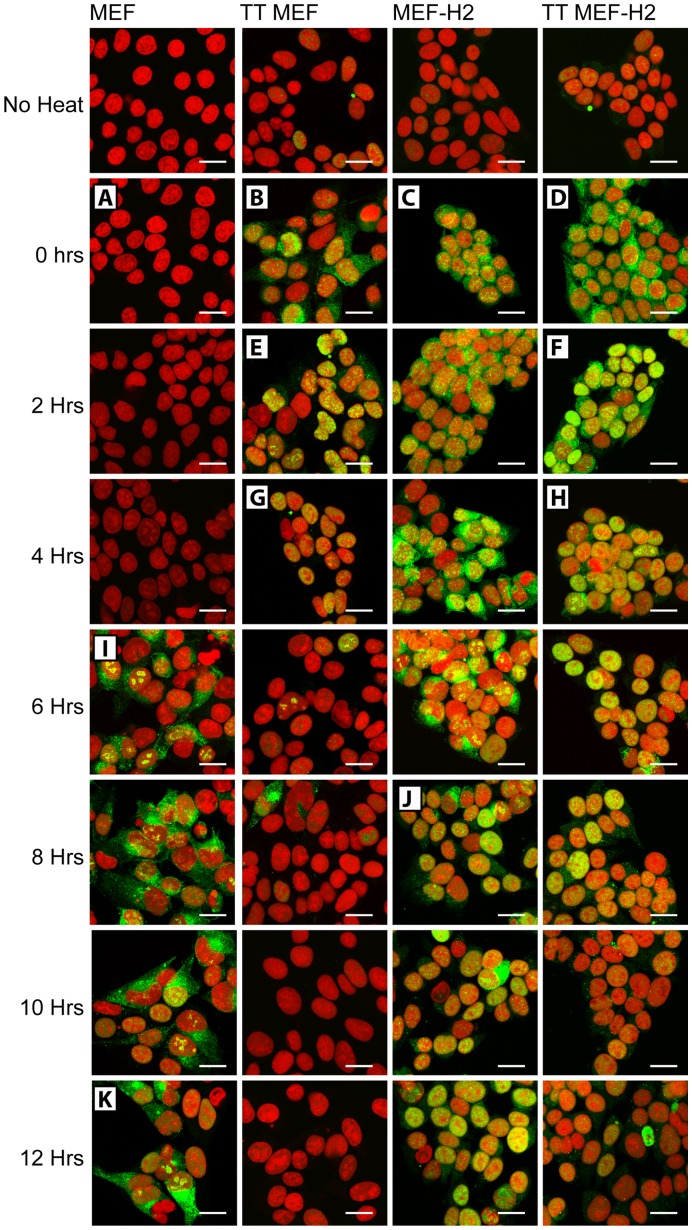
Hsp70.1 staining of digitonin permeabilized cells. Hsp70.1 staining of digitonin permeabilized cells. MEF, TT MEF, MEF-H2 & TT MEF-H2 heated at 44°C 20 min and harvested at specified time points after heating. Cells were permeabilized with digitonin prior to fixation and Hsp70.1 detected by immunofluorescence. Hsp70.1 (green), PI (red). Scale bars represent 20 µM. A – H mark critical events in the 8 hour time line.

At the same time, digitonin permeabilization was used to observe the accumulation and disaggregation of insoluble Hsp70.1 in MEF, TT MEF, MEF-H2 and TT MEF-H2 cells as they recovered from heat stress (Figure 5). TT MEF, MEF-H2 and TT MEF-H2 all contained significant amounts of insoluble Hsp70.1 immediately after heating (Fig. 5 B, C, D). No aggregated Hsp70.1 was detected in MEF immediately after heating as these cells did not contain Hsp70.1 at that time (Fig. 5A). The efficiency of how the four different cell types recovered from heat stress could be seen clearly in the rate at which insoluble Hsp70.1 was disaggregated and returned to being a soluble protein to the cytoplasm. TT MEF rapidly redistributed insoluble Hsp70.1 from the cytoplasm to the nucleus (Fig. 5E) and had returned almost all Hsp70.1 to the soluble cytoplasmic pool by 4 hours after heating (Fig. 5G). In contrast, MEF-H2, which are less resistant to heat induced apoptosis, responded more slowly and had not disaggregated all cytoplasmic Hsp70.1 until 8 hours after heating (Fig. 5J). TT MEF-H2 also recovered rapidly from heat, redistributing Hsp70.1 to the nucleus by 2 hours after heating (Fig. 5F) and clearing insoluble aggregates from the cytoplasm by 4 hours (Fig. 5H). Both MEF-H2 and TT MEF-H2 retained nuclear Hsp70.1 for an extended period after the cytoplasmic Hsp70.1 had been disaggregated (Fig. 5H & 5J). However, this protein was not insoluble, as shown by its ability to be extracted by 0.2% TX100 (Fig. S5.) and may be an artifact of the expression of a human Hsp70.1 transgene in these mouse cells.

MEF did not contain Hsp70.1 prior to heating and were highly sensitive to heat induced apoptosis. These cells did not start expressing Hsp70.1 until 6 hours after heating (Fig. S4). This newly expressed Hsp70.1 still formed insoluble aggregates similar to those seen in the other cell types (Fig. 5I), despite the heat stress occurring 6 hours prior to the arrival of the Hsp70.1 protein in the cell. These cytoplasmic and nucleolar aggregations of Hsp70.1 were still present at 12 hours (Fig. 5K) and persisted for up to 16 hours after heating (data not shown).

**Figure 6:**
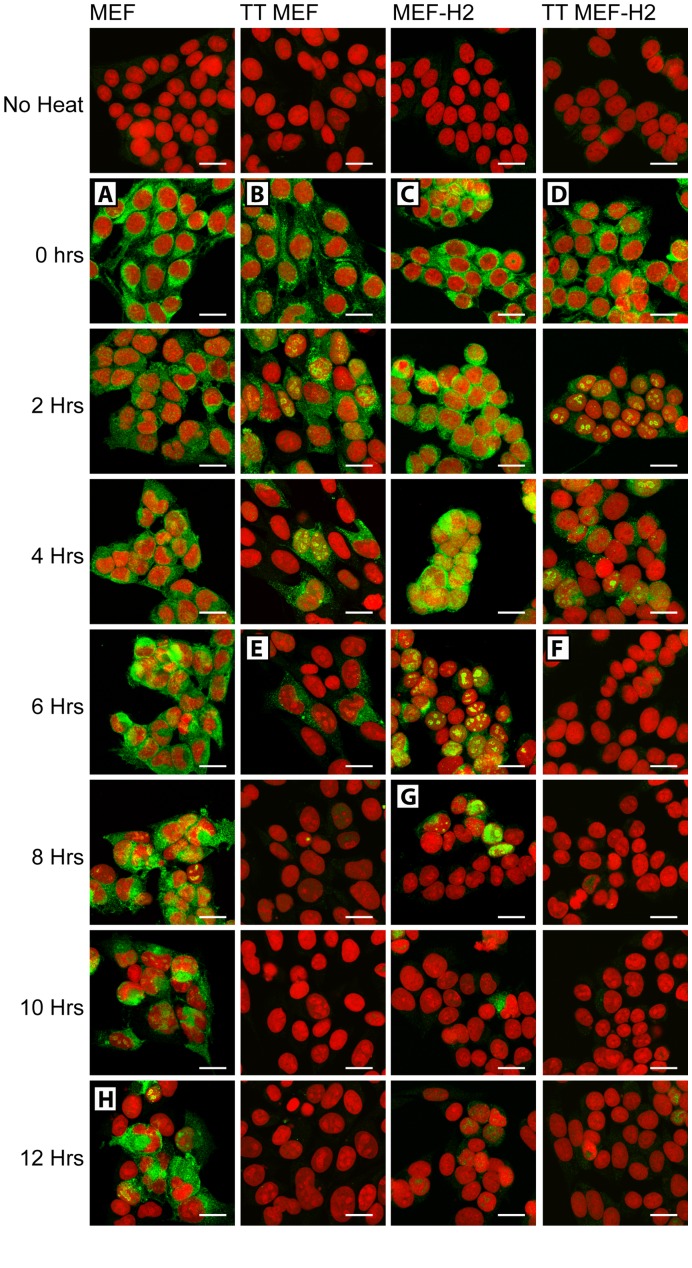
Hsc70 staining of digitonin permeabilized cells. Hsc70 staining of digitonin permeabilized cells. MEF, TT MEF, MEF-H2 & TT MEF-H2 heated at 44°C 20 min and harvested at specified time points after heating. Cells were permeabilized with digitonin prior to fixation and Hsc70 detected by immunofluorescence. Hsc70 (green), PI (red). Scale bars represent 20 µM. A – H mark critical events in the 8 hour time line.

The distribution of intracellular aggregates of Hsc70 was examined in an identical fashion (Fig. 6). Hsc70, which is expressed equally in all four cell types, illustrated their different responses to heat much more clearly. Heat induced rapid Hsc70 binding to insoluble cytoplasmic structures in all four cell types (Fig. 6 A, B, C, D), but in thermotolerant MEF and thermotolerant MEF-H2, most of the insoluble Hsc70 had been disaggregated by 6 hours after heating (Fig. 6E, F). MEF-H2 cells also disaggregated the insoluble Hsc70 but the protein was not fully extracted from digitonin permeabilized cells until 8 hours after heat treatment (Fig. 6G). Only in MEF did Hsc70 remain bound to insoluble cytoplasmic structures at 8 hours, persisting for up to 16 hours after heating (Fig. 6H & data not shown). While Hsc70 was initially restricted to the cytoplasm, it was also detected in the nucleoli of cells at later time points, although this nuclear Hsc70 was always less abundant than that found in the cytoplasm. It should also be noted that the expression of Hsp70.1 did not reduce the levels of Hsc70 fluorescence detected in cells immediately after heat (Fig. 6 A & C), indicating that the structures formed by Hsp70.1 and Hsc70 are independent of each other.

## Discussion

Despite the well-documented ability of Hsp70.1 to protect cells from heat induced cell death and to promote the repair of essential cell processes after heat damage, the molecular targets of Hsp70.1 responsible for these functions remain poorly defined. Previous studies have failed to identify an increased association of Hsp70.1 with soluble proteins from heat treated cells [39], [40], even though Hsp70.1 is known to bind to denatured proteins [26]. We hypothesized that Hsp70.1 may instead be binding to insoluble proteins within the cell, either associated with the cytoskeleton or intracellular membranes, that are usually discarded during preparation of the cytosol and thus excluded from standard biochemical analysis.

Our analysis has revealed that insoluble aggregates of Hsp70.1and Hsc70 are present in the cytoplasm and nucleus in large quantities. The cytoplasmic Hsp70.1/Hsc70 aggregates in particular are novel, as previous research has focused on the presence of Hsp70.1/Hsc70.1 in the nucleus of heated cells [23], [33]. To our knowledge, only Zeng et al. has reported the presence of cytoplasmic Hsp70.1 aggregates, although the technique he used (FRAP) failed to resolve these structures [41].

Our analysis of digitonin permeabilized cells has revealed a dynamic population of insoluble Hsp70.1/Hsc70 structures in heated cells. These structures are resistant to detergent extraction, indicating that they are not associated with internal cell membranes, yet show no resemblance to known intracellular protein networks. The binding of Hsp70.1/Hsc70 to these insoluble internal structures is both rapid and induced by low doses of heat, allowing the cell to respond to the first signs of protein damage. The speed and sensitivity of this response may be instrumental in protecting essential cell functions, although the exact mechanism of this protective function remains unknown.

Current understanding of Hsp70.1 and Hsc70 function would suggest that these insoluble complexes are formed by the binding of Hsp70.1/Hsc70 to heat denatured protein aggregates. Indeed, the insoluble Hsp70.1 structures that we observed formed in response to the proteotoxic stresses heat and sodium arsenite, but not to stresses that induce apoptosis but do not cause protein damage. However, the work of Kampinga et al. indicates that the formation of Hsp70 aggregates is independent of the aggregation of heat denatured proteins [33]. In our own study, Hsp70.1 also failed to form aggresome like structures with vimentin and the proteasome that are associated with denatured proteins [28]. Nor did we find any evidence that the insoluble Hsp70.1 was bound to other aggregated proteins, as chemical crosslinking of detergent extracted cells and purified Hsp70.1 gave identical Hsp70.1 crosslinking patterns.

Another hypothesis is that the Hsp70.1/Hsc70 is present in heat-induced stress granules that are formed from accumulated mRNAs, resulting in stalled translation [29] . However, confocal microscopy revealed a markedly different intracellular distribution of the stress granules and Hsp70.1 after heat (Fig. S3). Heated MEF recovered the ability to transcribe and translate Hsp70.1 that then went on to form stable aggregates, indicating that these structures can form in cells that are capable of efficient translation.

An alternative hypothesis, supported by the observation that chemically crosslinked Hsp70.1 from heated cells shows an identical electrophoretic mobility to crosslinked purified Hsp70.1, is that Hsp70.1 and Hsc70 polymerize in response to cell damage to form insoluble protein networks reminiscent of, but distinct from the cytoskeleton. While the oligomerization of Hsp70.1 and Hsc70 to form dimers and trimers in solution is well recognized [38], purified Hsc70 has also been reported to form insoluble polymeric complexes *in vitro* in the presence of ATP and YDJ1 [42]. We propose that a similar mechanism may be responsible for the rapid aggregation of Hsp70.1 and Hsc70 in response to proteotoxic stress and that these polymeric structures may be performing a structural role within cells or acting as a kind of scaffold for protein chaperone functions.

Although the true function of these structures remains unknown, the rate of Hsp70.1/Hsc70 disaggregation correlated with the rate of survival in MEF after heating. Thermotolerant cells that are almost completely resistant to a 20 minute 44°C heat dose disaggregated insoluble Hsp70.1/Hsc70 more rapidly than MEF-H2 cells that exhibit an intermediate rate of heat resistance. In contrast, MEF that do not express Hsp70.1 at the time of heating and are sensitive to heat induced apoptosis, show persistent Hsp70.1/Hsc70 aggregate formation for more than 16 hours after heating.

One important question arising from these results concerns the different roles of Hsp70.1 and Hsc70 in protecting cells from stress. Hsc70 is present in abundance in all cell types tested, but only those cells containing Hsp70.1 at the time of heating show increased resistance to thermal killing. This distinction in protein function could be due to the presence of Hsp70.1, but not Hsc70, in the nucleus during heating, where it might function to protect and repair transcription and other nuclear functions from heat induced damage [9]. Hsp70.1 and Hsc70 also appear to form different insoluble structures after heating.

The discovery of insoluble Hsp70.1/Hsc70 structures in the cytoplasm and nuclei of heated cells offers a new avenue for investigating the role of heat shock proteins in protecting cells from proteotoxic stress. These structures have been identified in a subfraction of cells that is all too often discarded and ignored by biochemical analysis. This oversight is largely due to practical considerations as the insoluble nature of these structures challenges our ability to study their function and composition without disrupting the structures themselves. These challenges will have to be overcome if we are to fully understand the function of these structures and how they are regulated by co-chaperones and post-translational modification.

## Materials and Methods

### Cell Culture and Reagents

MEF transformed with E1A and Ras were the kind gift of Dr Amato Giaccia (Stanford University, USA) [10]. HEK293 cells were supplied by the ATCC [43]. MEF and HEK293 were cultured in Dulbecco’s Modified Eagle Medium supplemented with 10% Serum Supreme (Cambrex), and 1% Penicillin/Streptomycin (Gibco BRL). Tissue culture vessels were heated by immersion in a circulating waterbath (Grant). Thermotolerance was induced by heating cells at 43°C for 15 min and allowing the cells to recover at 37°C for 8 hours after heating. Cells were irradiated in a UV crosslinker unit (UVP). The C92 (anti-HSP70.1) antibody was a kind gift of Dr William Welch (UCSF, CA) and the Hip antibody from Dr David Toft (Mayo Clinic, Boston MA). The anti-HSC70 (SPA-815) and anti-Hsp90 (SPA-830) antibodies were purchased from Stressgen, Hsp40 (Santa Cruz), HspBP1 (BD), Bag-1 (R&D), α-actin (Molecular Probes), Vimentin (AbCam), the Proteasome 20S subunit (Affinity Bioreagents) and eIF4E (Cell Signaling Technologies #9742). Sodium arsenite was purchased from Sigma.

### Apoptosis Assay

Apoptotic cells were scored by staining with PI and counting condensed nuclei by fluorescence microscopy, as described previously [20].

### Cell Fractionation

Cells grown in 6-well plates were incubated on ice with either 100 µg/ml digitonin or 0.2% TX100 in PBS for 5 minutes, and then washed gently with ice cold PBS. All liquid was aspirated and the remaining proteins harvested in Laemmli sample buffer. SDS-PAGE was conducted using the method of Laemmli [44]. Proteins were transferred to nitrocellulose (BioRad), blocked in milk proteins and incubated with antibodies. Fluorescent secondary antibodies (Molecular Probes) were detected using an Odyssey fluorescence scanner (Li-Cor). For chemical cross-linking, permeabilized cells were incubated in PBS with 1 mM dissuccinimidyl suberate (Pierce) for 10 min before harvesting into gel sample buffer.

### Confocal Microscopy

Cells were cultured on 18 mm coverslips. Cells were washed in PBS and fixed directly in 4% paraformaldehyde for 20 min, or permeabilized with 100 µg/ml digitonin or 0.2% TX100 in PBS or with RIPA buffer (50 mM Tris pH 8.0, 150 mM NaCl, 1% NP40, 0.5% Na deoxycholate, 0.1% SDS, 0.5 mM EDTA) on ice for 5 min. Permeabilized cells were washed in cold PBS and fixed as described. Fixed coverslips were permeabilized with 0.2% TX100, blocked in 4% BSA/PBS and incubated with antibodies diluted 1∶1000 for 3–4 hours on a rocking platform. Primary antibodies were detected with Alexa488 and Alexa590 labeled secondary antibodies (Molecular Probes). Images were captured with BioRad MRC 1024 laser scanning head equipped with a krypton/argon laser and attached to a Leica DMRBE microscope body with a 40x oil immersion lens (NA: 1.25). Z-sections were taken at 3 micron intervals, the images processed with Confocal Assistant 4.02 software and final image preparation with Adobe Photoshop.

## Supporting Information

Figure S1 **Hsp70.1 staining of permeabilized HEK293 & MEF-H2.** HEK293 cells were maintained at 37°C (A) or heated at 44°C for 20 min (B). Cells were permeabilized with 100 µg/ml digitonin and fixed with 4% paraformaldehyde. Hsp70.1 was detected by immunofluorescence (green) and the nuclei stained with PI (red). Hsp70.1 localization in MEF-H2 incubated with 0.1 mM Na arsenite for 20 min (C) and 60 min (D). Scale bars represent 20 µm.
(ZIP)

Figure S2 **Hsp70.1/Hsc70 does not co-localize with cytoskeletal proteins.** Hsp70.1 and Hsc70 do not co-localize with cytoskeletal proteins. All cells permeabilized with 100 µg/ml digitonin and analysed by immunofluorescence. Where indicated, cells were heated at 44°C for 20 min. Heated MEF-H2 stained for actin (A); tubulin (B). MEF-H2 double stained for vimentin (red C, D, E, F) and Hsp70.1 or Hsc70 (green): unheated/Hsp70.1 (C); heated/Hsp70.1 (D); unheated/Hsc70 (E); heated/Hsc70 (F). Scale bars represent 20 µm.
(ZIP)

Figure S3 **Hsp70.1 aggregates do not co-localize with stress granules.** MEF-H2 cells were heated at 44°C for 20 min and allowed to recover for 2 hours before some samples were permeabilized with 100 µg/ml digitonin as indicated. Nuclei were stained with PI (in white) while Hsp70.1 was detected with C92 and anti-mouse Alexa 488 (green). The stress granule marker eIF4E was detected with anti-eIF4E and anti-rabbit Alexa 647 (red). Scale bars represent 20 µm.
(ZIP)

Figure S4 **Hsp70.1 staining of non-permeabilized cells.** Hsp70.1 staining of non-permeabilized cells. MEF, TT MEF, MEF-H2 & TT MEF-H2 heated at 44°C 20 min and harvested at specified time points after heating. Cells were fixed immediately in formaldehyde and Hsp70.1 detected by immunofluorescence. Hsp70.1 (green), PI (red). Scale bars represent 20 µM.
(ZIP)

Figure S5 **Nuclear Hsp70.1 in MEF-H2.** Immediately after heating MEF-H2 were permeabilized with 100 µg/ml digitonin (A), 0.2% TX100 (B), or 8 hrs after heating permeabilized with 100 µg/ml digitonin (C), 0.2% TX100 (D). Hsp70.1 staining in white. The red outlines indicate cell nuclei. Scale bars represent 20 µM.(TIF)
(ZIP)
