# Supplementary figures and images for: Hsp70 Architecture: The Formation of Novel Polymeric Structures of Hsp70.1 and Hsc70 after Proteotoxic Stress
Source: PLoS One. 2012 Dec 19;7(12):e52351. doi: 10.1371/journal.pone.0052351 (PMC3526589; doi:10.1371/journal.pone.0052351)

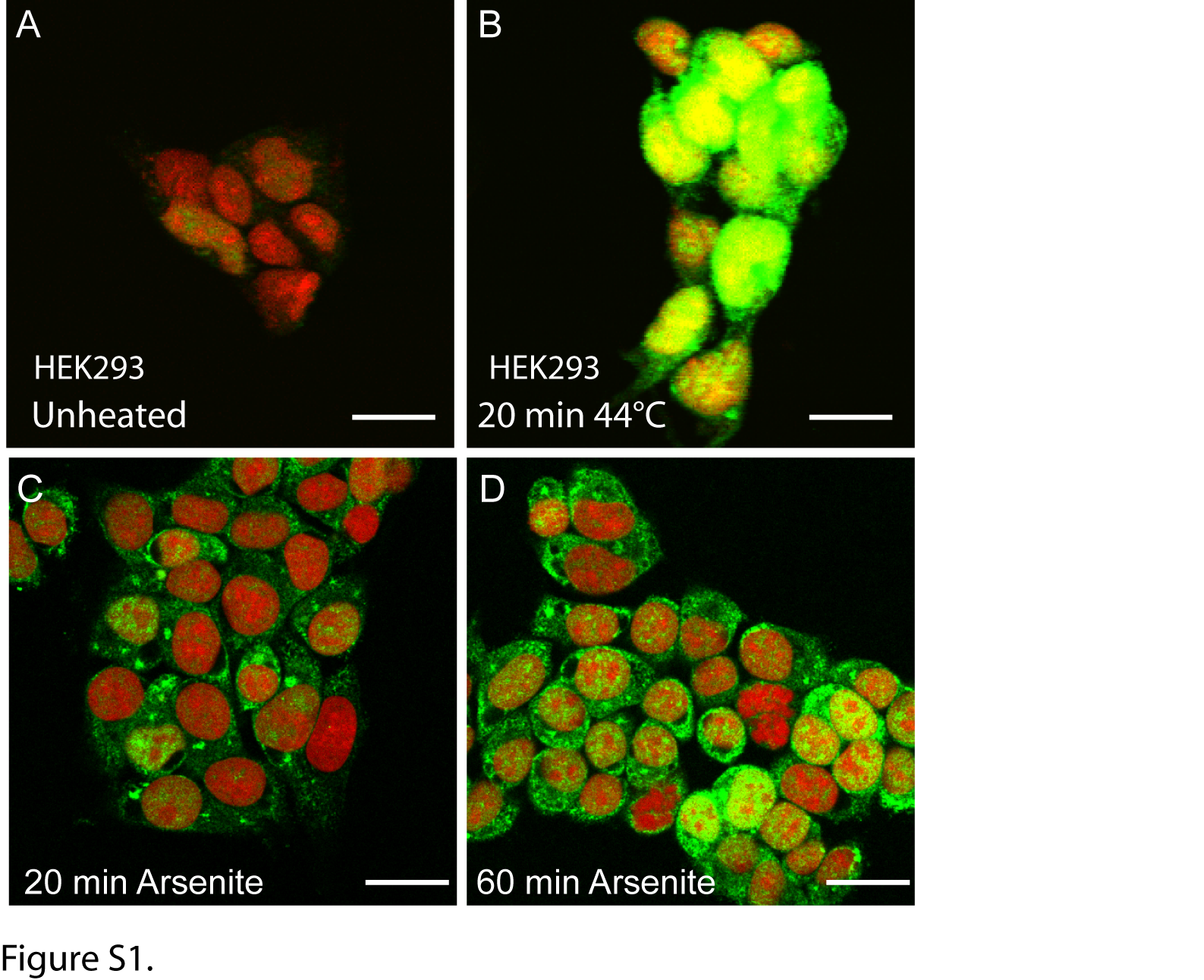

Supplement: Figure S1 — Hsp70.1 staining of permeabilized HEK293 & MEF-H2. HEK293 cells were maintained at 37°C (A) or heated at 44°C for 20 min (B). Cells were permeabilized with 100 µg/ml digitonin and fixed with 4% paraformaldehyde. Hsp70.1 was detected by immunofluorescence (green) and the nuclei stained with PI (red). Hsp70.1 localization in MEF-H2 incubated with 0.1 mM Na arsenite for 20 min (C) and 60 min (D). Scale bars represent 20 µm. (ZIP) [file pone.0052351.s001.zip › Figure S1.tif]

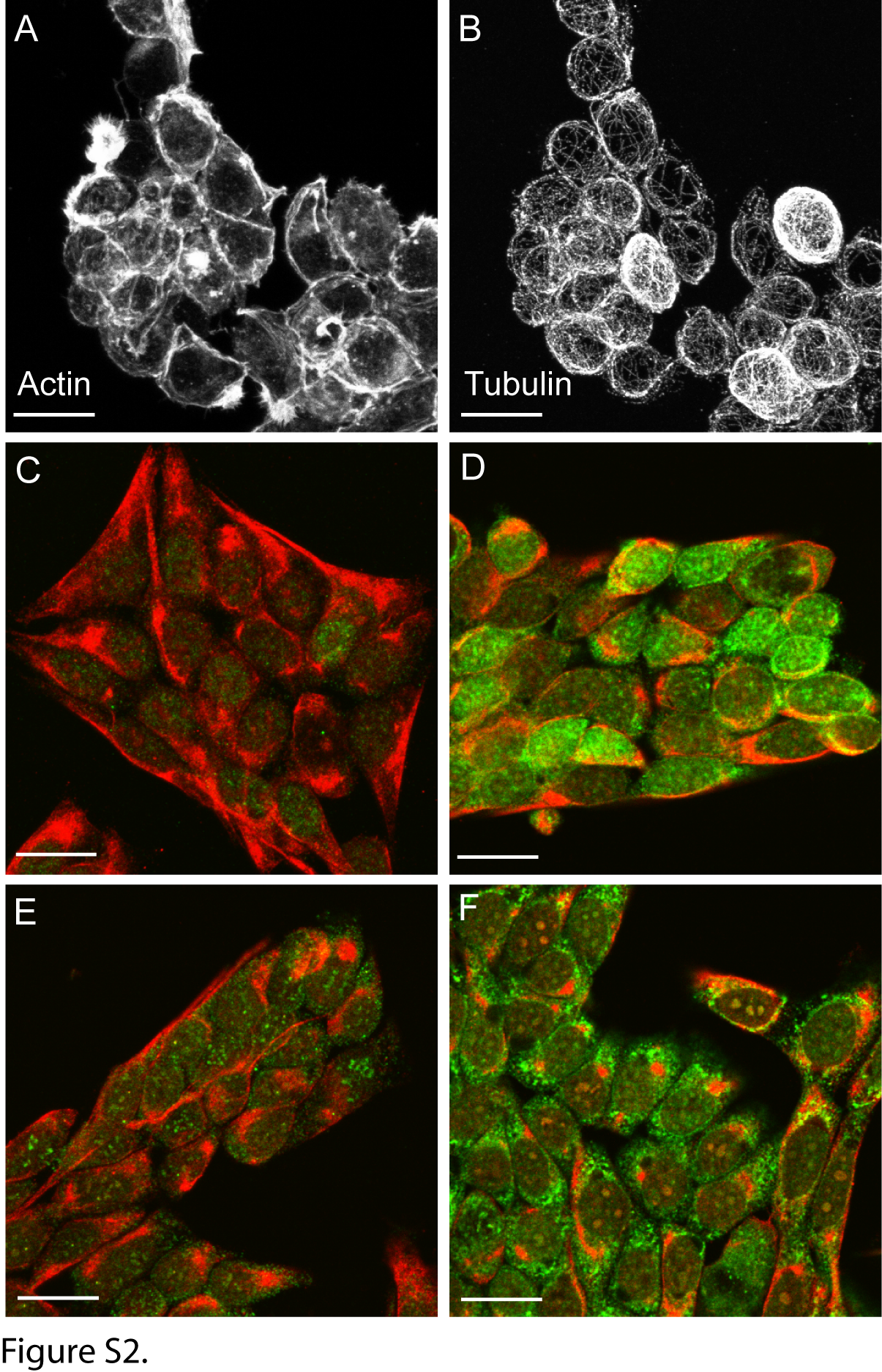

Supplement: Figure S2 — Hsp70.1/Hsc70 does not co-localize with cytoskeletal proteins. Hsp70.1 and Hsc70 do not co-localize with cytoskeletal proteins. All cells permeabilized with 100 µg/ml digitonin and analysed by immunofluorescence. Where indicated, cells were heated at 44°C for 20 min. Heated MEF-H2 stained for actin (A); tubulin (B). MEF-H2 double stained for vimentin (red C, D, E, F) and Hsp70.1 or Hsc70 (green): unheated/Hsp70.1 (C); heated/Hsp70.1 (D); unheated/Hsc70 (E); heated/Hsc70 (F). Scale bars represent 20 µm. (ZIP) [file pone.0052351.s002.zip › Figure S2.tif]

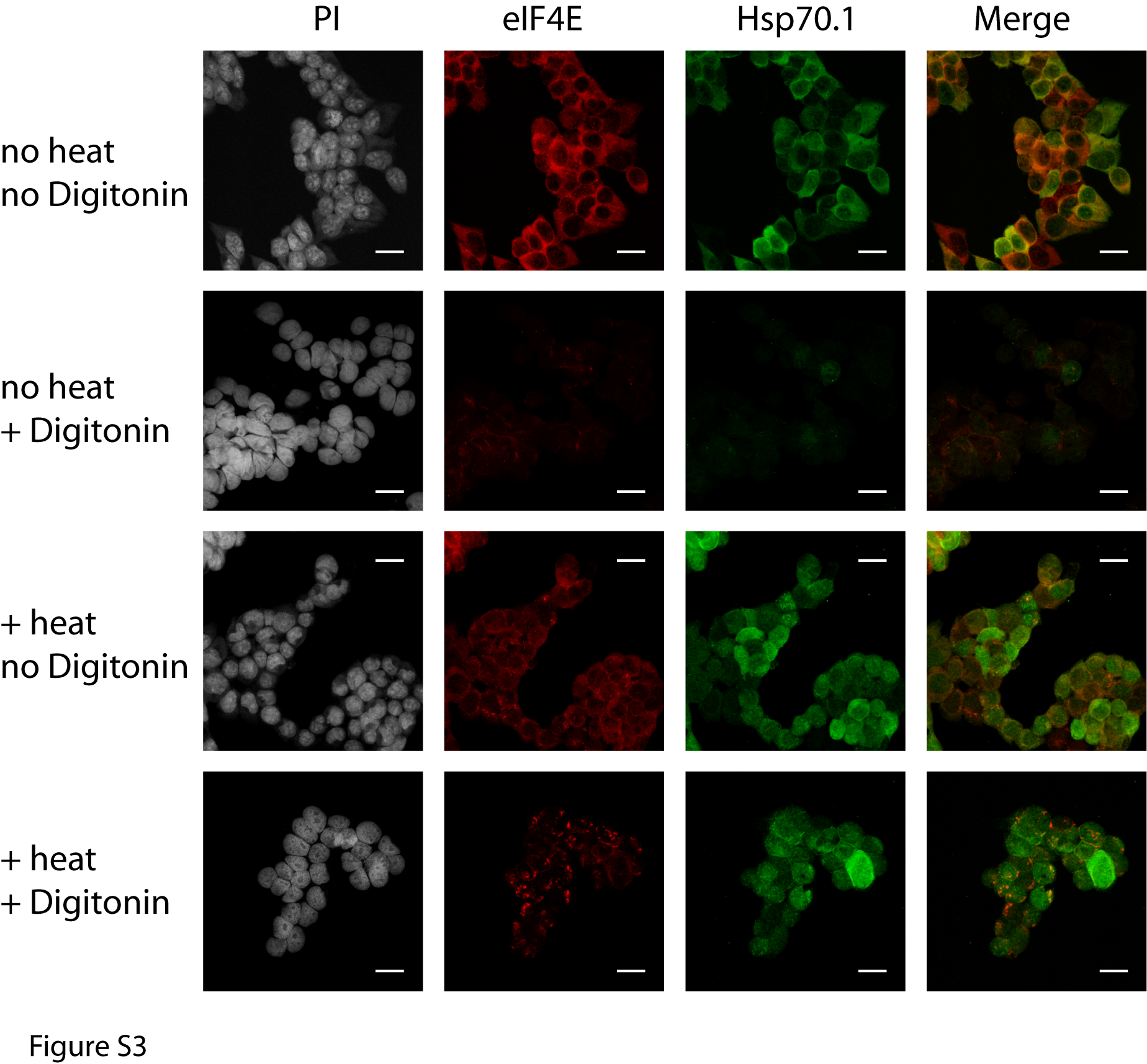

Supplement: Figure S3 — Hsp70.1 aggregates do not co-localize with stress granules. MEF-H2 cells were heated at 44°C for 20 min and allowed to recover for 2 hours before some samples were permeabilized with 100 µg/ml digitonin as indicated. Nuclei were stained with PI (in white) while Hsp70.1 was detected with C92 and anti-mouse Alexa 488 (green). The stress granule marker eIF4E was detected with anti-eIF4E and anti-rabbit Alexa 647 (red). Scale bars represent 20 µm. (ZIP) [file pone.0052351.s003.zip › Figure S3.tif]

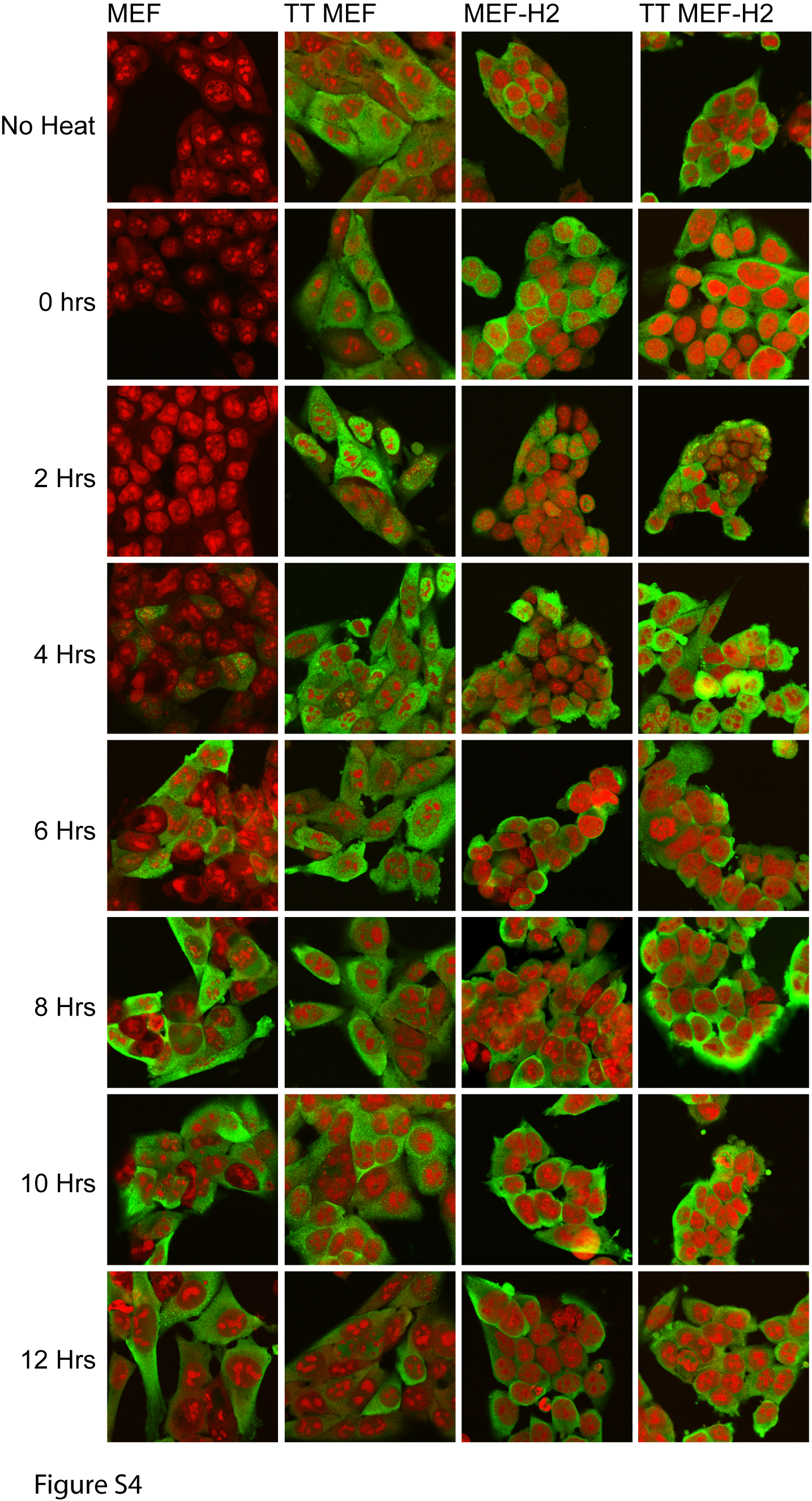

Supplement: Figure S4 — Hsp70.1 staining of non-permeabilized cells. Hsp70.1 staining of non-permeabilized cells. MEF, TT MEF, MEF-H2 & TT MEF-H2 heated at 44°C 20 min and harvested at specified time points after heating. Cells were fixed immediately in formaldehyde and Hsp70.1 detected by immunofluorescence. Hsp70.1 (green), PI (red). Scale bars represent 20 µM. (ZIP) [file pone.0052351.s004.zip › Figure S4.tif]

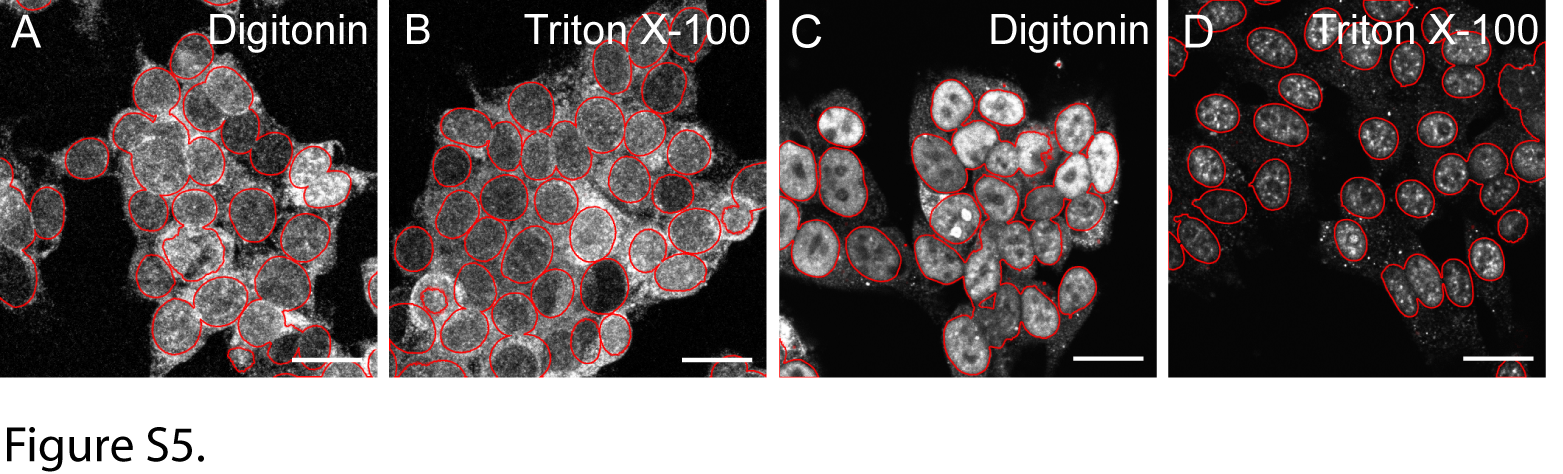

Supplement: Figure S5 — Nuclear Hsp70.1 in MEF-H2. Immediately after heating MEF-H2 were permeabilized with 100 µg/ml digitonin (A), 0.2% TX100 (B), or 8 hrs after heating permeabilized with 100 µg/ml digitonin (C), 0.2% TX100 (D). Hsp70.1 staining in white. The red outlines indicate cell nuclei. Scale bars represent 20 µM.(TIF) (ZIP) [file pone.0052351.s005.zip › Figure S5.tif]
